# Usage and positivity rates of Alzheimer's disease biomarkers in a memory clinic

**DOI:** 10.1002/alz.71442

**Published:** 2026-05-04

**Authors:** Anna Hofmann, Benjamin Saef, William J. B. Powell, Madeline Paczynski, Maria R. Ponisio, Clara Vila‐Castelar, Zachary Posey, Inez Y. Oh, Mackenzie R. Hofford, Melissa Aldinger, Randall J. Bateman, Cyrus A. Raji, Tammie L. S. Benzinger, Carlos Cruchaga, Alan Dow, Rachel Buckley, Chengjie Xiong, John C. Morris, B. Joy Snider, Aditi Gupta, Suzanne E. Schindler

**Affiliations:** ^1^ Department of Neurology Washington University St. Louis Missouri USA; ^2^ Knight Alzheimer Disease Research Center Washington University St. Louis Missouri USA; ^3^ Institute for Informatics, Data Science and Biostatistics Washington University St. Louis Missouri USA; ^4^ Mallinckrodt Institute of Radiology Washington University Medicine St. Louis Missouri USA; ^5^ Department of Psychiatry Washington University Medicine St. Louis Missouri USA; ^6^ BJC Health St. Louis Missouri USA; ^7^ Massachusetts General Research Institute Harvard Medical School Charlestown Massachusetts USA

**Keywords:** amyloid‐targeting treatments, biomarkers, racial differences, real‐world evidence, sex differences

## Abstract

**INTRODUCTION:**

Usage of biomarker tests for Alzheimer's disease pathology and rates of positivity were assessed at the Washington University Memory Diagnostic Center.

**METHODS:**

Patients who underwent at least one biomarker test for clinical purposes between June 2021 and March 2025 were included (*n* = 1136). Data were retrospectively extracted from electronic health records.

**RESULTS:**

The median age was 73.2 years (52% female; 93% White). In total, 455 amyloid positron emission tomography (PET) scans, 505 cerebrospinal fluid tests, and 242 blood tests were performed. The number of biomarker tests increased seven‐fold over the past 4 years. The rate of positivity was ≈70% across modalities. Higher rates of biomarker positivity were associated with older age, female sex, and White race; lower rates were associated with hypertension and diabetes.

**DISCUSSION:**

Biomarker testing greatly increased following the approval of amyloid‐targeting treatments. The overall rate of biomarker positivity was high and varied by demographic factors and medical comorbidities.

## Highlights

- Alzheimer's disease biomarker testing increased seven‐fold over four years
- AD biomarker positivity was associated with older age, female sex, and White race
- AD biomarker negativity was associated with hypertension and diabetes

## 1 BACKGROUND

Alzheimer's disease (AD) is characterized by amyloid plaques and tau neurofibrillary tangles, and may cause progressive cognitive decline leading to dementia.^1^ Even after a comprehensive clinical evaluation, mild cognitive impairment (MCI) or dementia due to AD (termed symptomatic AD) is frequently misdiagnosed unless biomarker testing is performed to assess for the presence of AD pathology.^2^, ^3^ Furthermore, evidence of amyloid pathology is required for initiation of the amyloid‐targeting treatments (ATTs), lecanemab and donanemab, which have received traditional U.S. Food and Drug Administration (FDA) approval for early symptomatic AD.^4^, ^5^ Therefore, biomarker testing is essential to accurately diagnose AD as the likely cause of cognitive impairment and to determine whether patients may be eligible for ATTs.

Three different biomarker modalities are typically used in the clinic to confirm the presence of amyloid pathology: amyloid positron emission tomography (PET) scans, cerebrospinal fluid (CSF) tests, and blood tests.^6^, ^7^, ^8^ Amyloid PET and CSF tests are well established and have high performance in the detection of amyloid pathology with different specific advantages.^9^ However, amyloid PET imaging is expensive, involves the use of radioactive tracers, and requires highly specialized equipment and personnel.^7^ CSF is collected via a lumbar puncture, which is invasive and must be performed by a skilled clinician.^8^ Fortunately, recent advances have yielded highly accurate AD blood tests that are now clinically available.^6^, ^10^, ^11^ Compared to amyloid PET and CSF tests, AD blood tests are less invasive, easily accessible, and highly scalable, making them ideally suited for clinical applications that may enable accurate diagnosis of AD in the broader population. In May 2025, the Fujirebio Lumipulse plasma phosphorylated tau 217 (p‐tau217)/amyloid beta (Aβ) 42 test was the first AD blood test to receive FDA clearance. Given its high diagnostic accuracy and regulatory approval, a positive result on this test is sufficient to confirm amyloid pathology and to initiate ATTs per appropriate use recommendations.^12^ Other AD blood tests, including PrecivityAD2, are clinically available as laboratory developed tests.^13^ The PrecivityAD2 test combines the Aβ42/40 and p‐tau217/non‐phospho‐tau217 ratios into an amyloid probability score that stratifies the likelihood of amyloid pathology.^14^

AD biomarkers have primarily been studied in highly‐selected individuals participating in research studies.^10^ However, patients in real‐world clinical cohorts are typically thought to be older, less educated, more ethno‐racially diverse, and have higher rates of comorbidities and medication usage.^15^ There have been inconsistent results concerning whether AD biomarkers vary by sex.^16^ One study found higher rates of amyloid PET positivity and higher amyloid burden in females compared to males with cognitive impairment.^17^ However, other studies have found higher concentrations of plasma biomarkers of AD pathology, such as plasma p‐tau181 and p‐tau217, in men compared to women.^16^, ^18^ There may even be sex differences in how these fluid biomarkers are associated with downstream markers of tau accumulation, neurodegeneration, and cognition.^19^, ^20^, ^21^, ^22^, ^23^, ^24^ In addition, there is uncertainty about whether AD biomarkers vary by race. Previous PET studies have reported lower rates of biomarker positivity and/or lower continuous amyloid levels in Black compared to White patients,^25^, ^26^ although other studies reached opposite or uncertain results.^27^, ^28^ A lower frequency of AD biomarker positivity for Black compared to White individuals was also described for CSF^29^, ^30^ and blood tests,^31^, ^32^, ^33^ although these results are also inconsistent.^34^, ^35^, ^36^ Thus, the associations between AD biomarkers, demographic factors, and medical comorbidities may be complex, and further examination in real‐world settings may be informative.

The Washington University (WashU) Memory Diagnostic Center (MDC) in St. Louis, Missouri, sees ≈4000 patients annually with memory and thinking concerns who are typically referred by their primary care providers. In recent years, MDC clinicians have been able to choose the biomarker modality that is optimal for their patient to increase diagnostic accuracy and to evaluate eligibility for ATTs.^3^, ^13^ The numbers of each biomarker test performed over time were examined both before and after the traditional FDA approval of lecanemab in July 2023. Furthermore, the rates of biomarker positivity were examined as a function of age, race, sex, and medical comorbidities.

**RESEARCH IN CONTEXT**

1. **Systematic review**: PubMed was utilized to screen for original research articles investigating differences in Alzheimer's disease (AD) biomarker test results by sex, race, and medical comorbidities. Some evidence from research cohorts suggests there are differences in AD biomarker positivity rates associated with demographic factors and medical comorbidities, although results are inconsistent.
2. **Interpretation**: In this real‐world cohort from a memory clinic, a higher rate of AD biomarker positivity was associated with older age, female sex, and White race, while a lower rate was associated with hypertension and diabetes. The expected likelihood of biomarker positivity based on demographic factors and medical comorbidities may be considered when interpreting AD biomarker testing in clinical practice.
3. **Future directions**: These findings should be further investigated in larger, more ethno‐racially diverse cohorts from multiple clinical settings.

## 2 Methods

### 2.1 Clinical infrastructure and patient population

The MDC is the faculty outpatient clinical practice affiliated with WashU and receives referrals from a large catchment area primarily located in Missouri and Illinois.^37^ The clinical and demographic data from consecutive patients who underwent AD biomarker testing as part of clinical care between June 2021 and March 2025, were retrospectively collected from electronic health records.

### 2.2 Data extraction from electronic health records

Clinical variables were automatically extracted from electronic health records: demographics (age, sex, race, ethnicity, and body mass index [BMI]); medical comorbidities based on diagnostic codes (hypertension, hyperlipidemia, diabetes, cerebrovascular disease, myocardial infarction, chronic kidney disease [CKD], liver cirrhosis, and polyneuropathy); laboratory values (creatinine, estimated glomerular filtration rate [eGFR], glycosylated hemoglobin [HbA1c], low density lipoprotein [LDL], and triglycerides); and clinical scores (Mini‐Mental State Examination [MMSE] and Clinical Dementia Rating [CDR]). Race and ethnicity were self‐reported by patients. Lecanemab treatment status was determined via manual chart review of clinic records. All longitudinal data were selected from the date closest in time to the corresponding clinical biomarker test, and only data within 2 years of the test were included.

### 2.3 AD biomarker testing

Only biomarker data from tests performed as part of clinical care, and occurring prior to lecanemab treatment, were included. At WashU MDC, amyloid PET is routinely performed using Florbetaben or Florbetapir tracers, which are interpreted visually as either positive or negative for cortical amyloid retention. Centiloid (CL) quantification is increasingly used as a standardized, quantitative adjunct to visual assessment. CL values are derived from standardized uptake value ratios (SUVRs) and provide a continuous measure of amyloid burden on a scale anchored at 0 for young controls and 100 for patients with mild‐to‐moderate AD dementia. For this study, CL quantification was performed retrospectively using MIM software, with each scan analyzed individually rather than through an automated processing pipeline.^38^

CSF testing was performed with the Roche Elecsys p‐tau181/Aβ42 first‐generation test performed at Mayo Clinic before June 2023, the second‐generation test performed at Mayo Clinic between June 2023 and June 2024, and the second‐generation test performed at WashU after June 2024.^39^

AD blood tests started being performed in February 2022 at C2N Diagnostics with the PrecivityAD test, which transitioned to the PrecivityAD2 test after July 2023. The PrecivityAD test includes the Aβ42/40 ratio, age, and apolipoprotein E (*APOE*) proteotype to generate an amyloid probability score (APS).^40^ The PrecivityAD2 test combines the Aβ42/40 and p‐tau217/non‐phospho‐tau217 ratios to generate the amyloid probability score (APS2).^14^ Both versions of this blood test utilize a C2N proprietary mass spectrometry platform.

### 2.4 Statistical analyses

The characteristics of patients at their first biomarker test were examined and the statistical significance of differences between groups was assessed using chi‐square (*χ* ^2^) tests for categorical variables and Kruskal–Wallis tests for continuous variables. To assess the effects of demographic and clinical factors on biomarker status (positive or negative), a logistic regression analysis was performed with biomarker status as the outcome, and with predictors of age, sex, race (Black or White), and presence of different comorbidities (hypertension, diabetes, CKD, hyperlipidemia, and cerebrovascular disease). Comorbidities that affected less than 10% of patients in our cohort were not included in the model to maintain statistical power. For individuals with multiple biomarker tests, only the most recent test (and therefore most likely to be definitive) and associated information was included in the logistic regression model. Statistical analyses were performed with R version 4.3.1. Plots were created with GraphPad Prism v.10.4.0 or using the ggplot2 package in R.

### 2.5 Ethics

The study was approved by the WashU Institutional Review Committee. It was performed in accordance with the 1964 Declaration of Helsinki and its later amendments or comparable ethical standards.

### 2.6 Data access and availability

Because data in this study were from the health records of clinic patients who have not consented to data sharing, individual level clinical data are not readily sharable. However, anonymized data derived from MDC electronic health records can be requested from https://i2db.wustl.edu/consultation‐services/.

## 3 RESULTS

### 3.1 Patient demographics and treatment numbers

**FIGURE 1:**
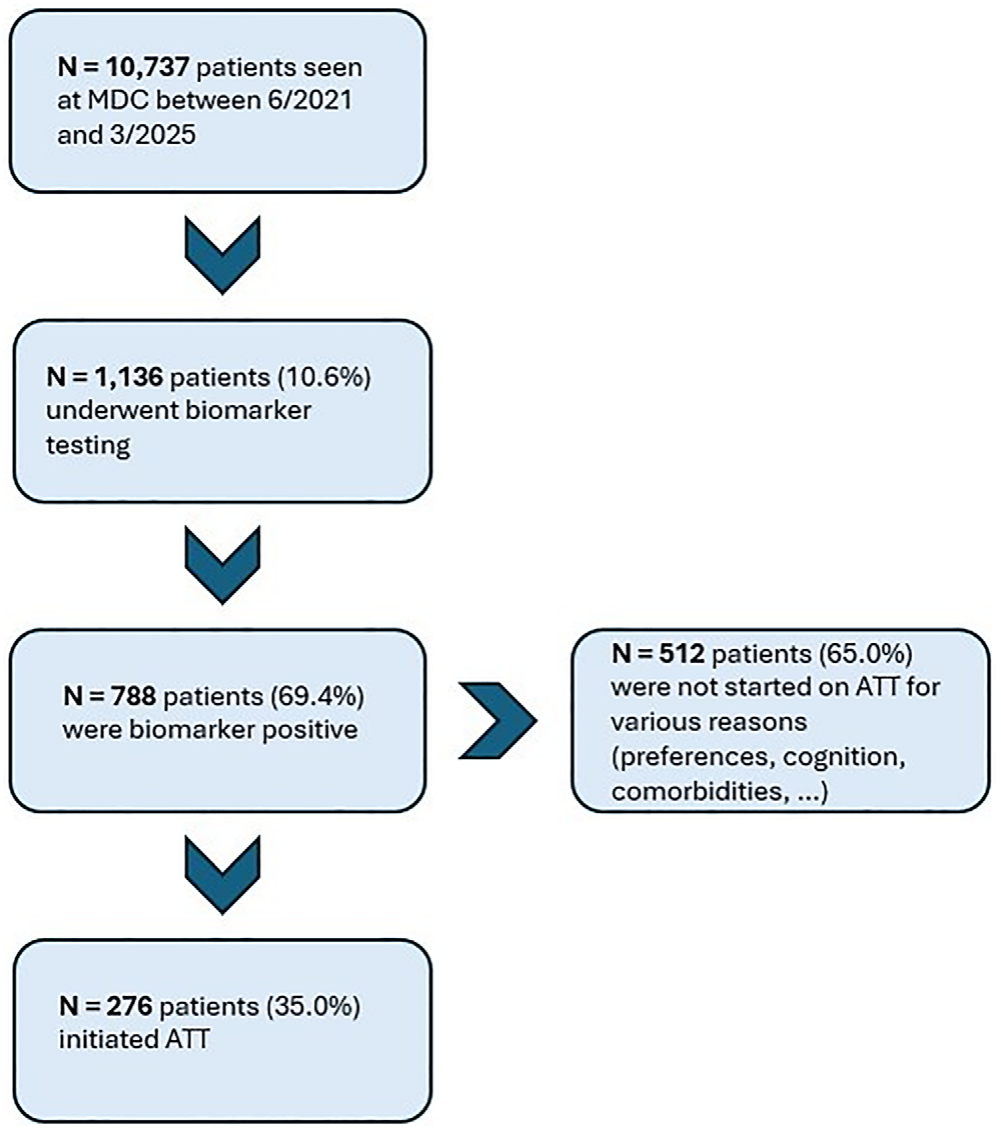
Consort diagram illustrating the patients’ journey from MDC visit until potential ATT start between 6/2021 and 3/2025. ATT, amyloid‐targeting treatment; Memory Diagnostic Clinic.

Between July 2021 and March 2025, a total of 10,737 patients were seen at MDC. Of those, 1136 (10.6%) underwent AD biomarker testing with at least one modality. Notably, a major reason for biomarker testing was to confirm amyloid pathology in patients who were potential ATT candidates. Of the 788 patients who were AD biomarker positive (69.4%), 276 (35.0%) subsequently started treatment with lecanemab (Figure 1).

The characteristics for the overall cohort of interest, that is, those who underwent at least one AD biomarker test as part of their clinical care (*n* = 1,136), at the timepoint of their first biomarker test and stratified by test result, are shown in Table 1. The median patient age was 73.2 years; 52% were female, 93% were White, and 99% were non‐Hispanic. Only 53 individuals undergoing AD biomarker testing were Black (5%). The median BMI was 26 kg/m^2^ (overweight). Hypertension and hyperlipidemia were common (63% and 62%, respectively); nearly 20% of patients had diabetes and/or reported cerebrovascular disease, about 10% had CKD and/or polyneuropathy, and about 5% had a history of myocardial infarction. Median laboratory values including creatinine, eGFR, HbA1c, LDL, and triglycerides were within the normal range across the entire cohort. Only 22 (2%) of all patients were cognitively unimpaired according to the CDR global score. Most patients (764, 67%) had MCI or very mild dementia (CDR 0.5), whereas some (195, 17%) had mild, moderate, or severe dementia (CDR ≥1). The median MMSE score was 25. Of all patients who underwent AD biomarker testing, 276 (24%) subsequently initiated treatment with lecanemab.

**TABLE 1:** Characteristics of all patients undergoing AD biomarker testing, stratified by test result.

Characteristic	*n*	All(*n* = 1136)	*n*	Biomarker positive (*n* = 788)	*n*	Biomarker negative(*n* = 348)	*p*‐value
Age, years	1136	73.2 (68.4–78.2)	788	73.5 (69.2−78.4)	348	71.8 (66.4−78)	<0.01
Sex, female	1136	590 (51.9%)	788	436 (55.3%)	348	154 (44.3%)	<0.001
Race, Black/White/other	1120	53/1053/14	778	27/744/7	342	26/309/7	<0.01
Ethnicity, non‐Hispanic	1107	1094 (98.8%)	765	755 (98.7%)	342	339 (99.1%)	0.54
Lecanemab treatment	1136	276 (24.3%)	788	276 (35.0%)	348	0 (0.0%)	<0.0001
BMI, kg/m^2^	544	26 (23–29.7)	372	25.2 (22.4−29.2)	172	27.4 (24.8−31.3)	<0.0001
Hypertension	1070	673 (62.9%)	749	445 (59.4%)	321	228 (71.0%)	<0.001
Hyperlipidemia	1070	667 (62.3%)	749	462 (61.7%)	321	205 (63.9%)	0.5
Diabetes	1070	213 (19.9%)	749	118 (15.8%)	321	95 (29.6%)	<0.0001
Cerebrovascular disease	1070	198 (18.5%)	749	123 (16.4%)	321	75 (23.4%)	<0.01
Myocardial infarction	1070	58 (5.4%)	749	33 (4.4%)	321	25 (7.8%)	0.03
Chronic kidney disease	1070	119 (11.1%)	749	6 (9.1%)	321	51 (15.9%)	<0.01
Liver cirrhosis	1070	5 (0.5%)	749	4 (0.5%)	321	1 (0.3%)	0.62
Polyneuropathy	1070	103 (9.6%)	749	60 (8.0%)	321	43 (13.4%)	<0.01
Creatinine, 0.6–1.1 mg/dL	465	0.93 (0.8−1.16)	302	0.9 (0.8−1.06)	163	1.0 (0.83−1.24)	<0.001
GFR, ≥90 mL/min/1.73 m^2^)	410	74 (60−85)	263	75 (62‐86)	147	72 (54‐82)	0.02
HbA1c, 4.0%–5.6%	173	5.9 (5.5−6.4)	102	5.8 (5.5‐6.3)	71	5.9 (5.5‐6.4)	0.86
LDL, ≤129 mg/dL	291	84 (63−112)	192	87 (67‐116)	99	74 (56‐100)	<0.01
Triglycerides, ≤149 mg/dL	300	94 (69−138)	198	92 (70‐132)	102	103 (69‐145)	0.24
MMSE score	1024	25 (22−27)	715	24 (21‐26)	309	26 (23‐28)	<0.0001
CDR Global Score, 0/0.5/≥1	981	22/764/195	698	10/552/136	283	12/212/59	0.02

*Note*: Age, laboratory values, BMI, and cognitive scores are selected at the time of first biomarker test. Normative values and units are indicated in parentheses for laboratory values. Some individuals did not identify as Black or White. Information on ethnicity, BMI, comorbidities, laboratory values and cognition were not available for every individual; the “*n*” columns indicate the number of patients for which the data were available. Counts and percentages are provided for categorical variables. Median and interquartile range are indicated for continuous variables. For the classification of either biomarker “positive” or “negative,” in case of multiple biomarker testing, the last biomarker test result was used. Notably, there were *n* = 8 patients with a PrecivityAD test only with an “intermediate” result. Those are listed as “negative” here. A chi‐square (*χ* ^2^) test was calculated for categorial variables and a Kruskal–Wallis test for continuous variables.

Abbreviations: AD, Alzheimer's disease; BMI, body mass index; GFR, glomerular filtration rate; HbA1c, hemoglobin A1c; LDL, low‐density lipoprotein; MMSE, Mini‐Mental State Examination; CDR, Clinical Dementia Rating.

There were significant differences between the AD biomarker positive and negative groups: AD biomarker positive patients were older (*p* < 0.01) and more likely to be female (*p* < 0.001). Race was also associated with biomarker status (*p* < 0.01). AD biomarker‐positive patients also had a lower BMI (*p* < 0.0001), and were less likely to have hypertension (*p* < 0.001), diabetes (*p* < 0.0001), cerebrovascular disease (*p* < 0.01), myocardial infarction (*p* = 0.03), CKD (*p* < 0.01), or polyneuropathy (*p* < 0.01). AD biomarker‐positive patients had a lower median creatinine (*p* < 0.001), and higher eGFR ( = 0.02) and LDL (*p* < 0.01). The median MMSE score was lower in the AD biomarker positive group (*p* < 0.0001). Also, CDR was associated with AD biomarker status (*p* = 0.02). Only AD biomarker positive patients were treated with lecanemab (*p* < 0.0001).

### 3.2 Demographic and clinical differences between biomarker groups

The same information shown in Table 1, but stratified by the three different modalities for patients who underwent testing with a single biomarker only, is shown in Table S1. Most patients underwent a CSF test only (456, 40%) or an amyloid PET scan only (414, 36%), whereas 203 patients (18%) received an AD blood test only. Sex, race, ethnicity, BMI, and the proportion of patients treated with lecanemab, were evenly distributed across modalities. Age was associated with biomarker modality chosen (*p *< 0.0001) with the following median ages: 73.8 years for amyloid PET, 71.8 years for CSF tests, and 75.4 for blood tests. Both CDR global and MMSE scores were associated with the biomarker modality (*p *< 0.0001), with the following percentages of patients with dementia (CDR ≥1): 12% for amyloid PET, 23% for CSF tests, and 26% for blood tests. Cerebrovascular disease (*p *= 0.01), LDL concentration (*p* = 0.003), and CKD (*p *= 0.046) were also associated with the biomarker modality chosen. All other comorbidities and laboratory values were evenly distributed between the three different biomarker modalities.

### 3.3 Demographic and clinical differences between single versus multiple biomarker testing

Table S2 shows the characteristics of patients at their first biomarker test as provided in Table 1, but stratified by whether patients underwent either a single or multiple biomarker tests. Few patients (63, 6%) underwent AD biomarker testing with more than one modality. Of those, 24 received amyloid PET and CSF testing; 22 CSF and blood testing; 14 amyloid PET and blood testing, and 3 amyloid PET, CSF, and blood testing. Demographic details, BMI, and cognition were evenly distributed between the two groups. Cerebrovascular disease was more common among patients with single biomarker testing (*p* = 0.01), but the frequency of myocardial infarction (*p* = 0.03) and median LDL levels in blood (*p* = 0.02) were higher in those patients who underwent testing with multiple biomarkers.

### 3.4 Biomarker testing volume

**FIGURE 2:**
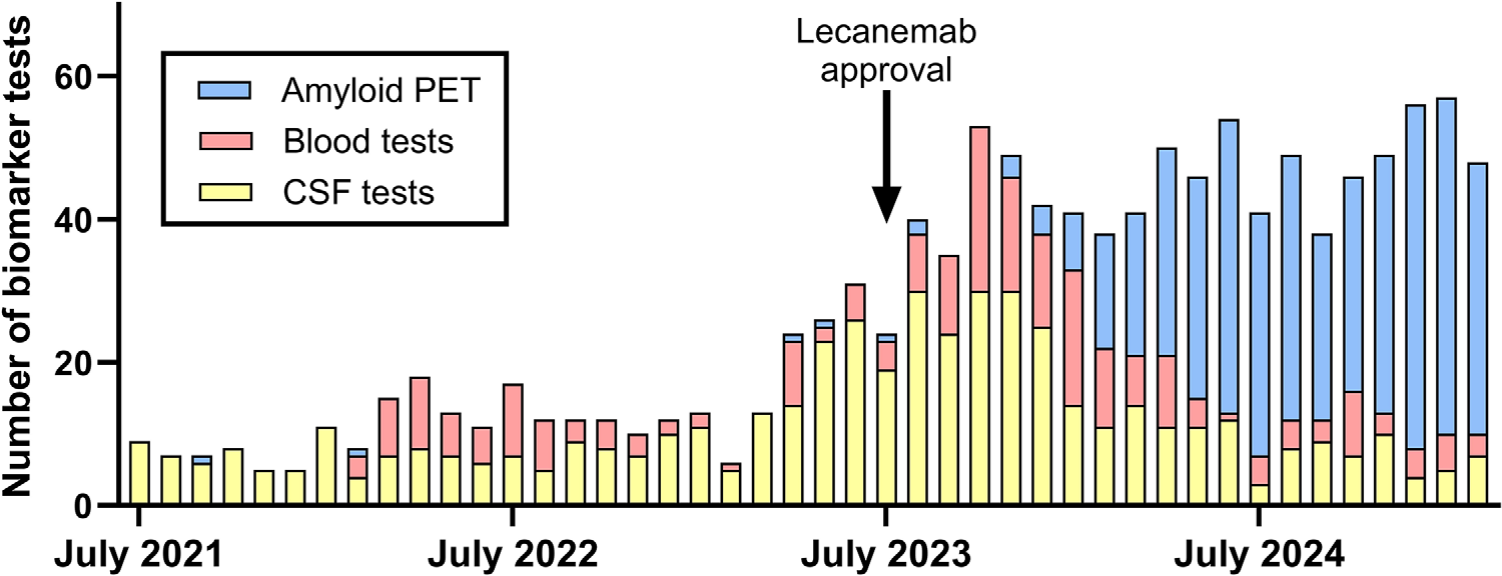
Monthly numbers of biomarker tests over time. Number of amyloid PET, CSF, and blood tests performed per month at WashU MDC between July 2021 and March 2025. CSF, cerebrospinal fluid; MDC, Memory Diagnostic Clinic; PET, positron emission tomography; WashU, Washington University.

The monthly volume of biomarker testing increased from ≈7 tests per month in 2021, to ≈50 tests per month after July 2024 (Figure 2). Blood tests started being performed in February 2022. Between July 2021 and March 2025, among the 1136 patients who received at least one AD biomarker test at MDC, the numbers of tests performed were as follows: 455 amyloid PET scans, 505 CSF tests, 155 PrecivityAD2 blood tests, and 87 PrecivityAD blood tests (Tables S3 and S4). As mentioned, some individuals underwent multiple tests.

### 3.5 Biomarker test results and comorbidity rates by demographic factors

**FIGURE 3:**
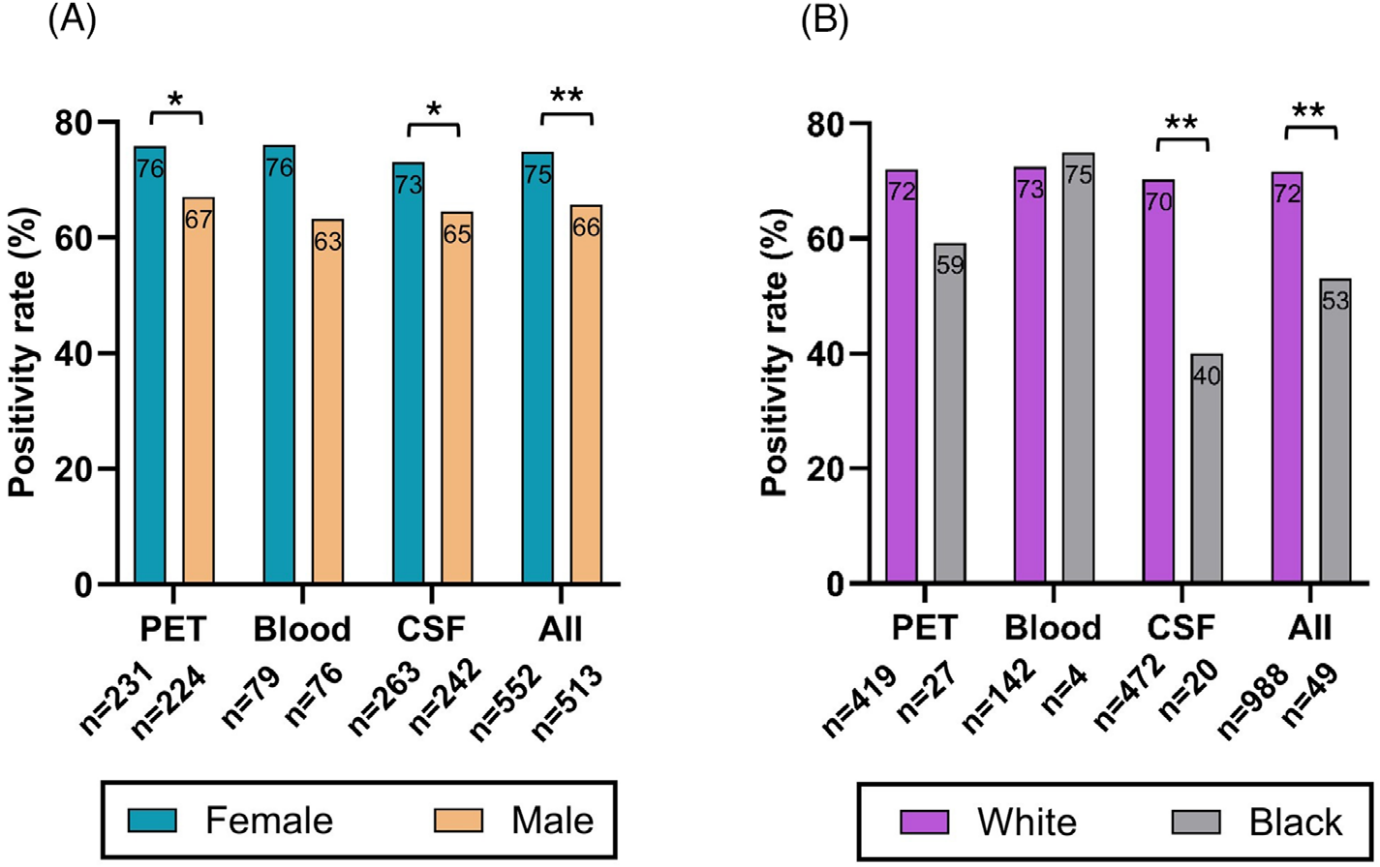
Rates of biomarker positivity stratified by sex (a) and race (b). (See Tables S3 and S4 for detailed information on test results for each group.) Blood tests and the overall biomarker positivity count include Precivity AD2 only. Note that 63 patients underwent multiple biomarker testing. For the overall biomarker positivity count, only the last test performed was included. Chi‐square (*χ* ^2^) tests have been calculated for group comparisons.

**FIGURE 4:**
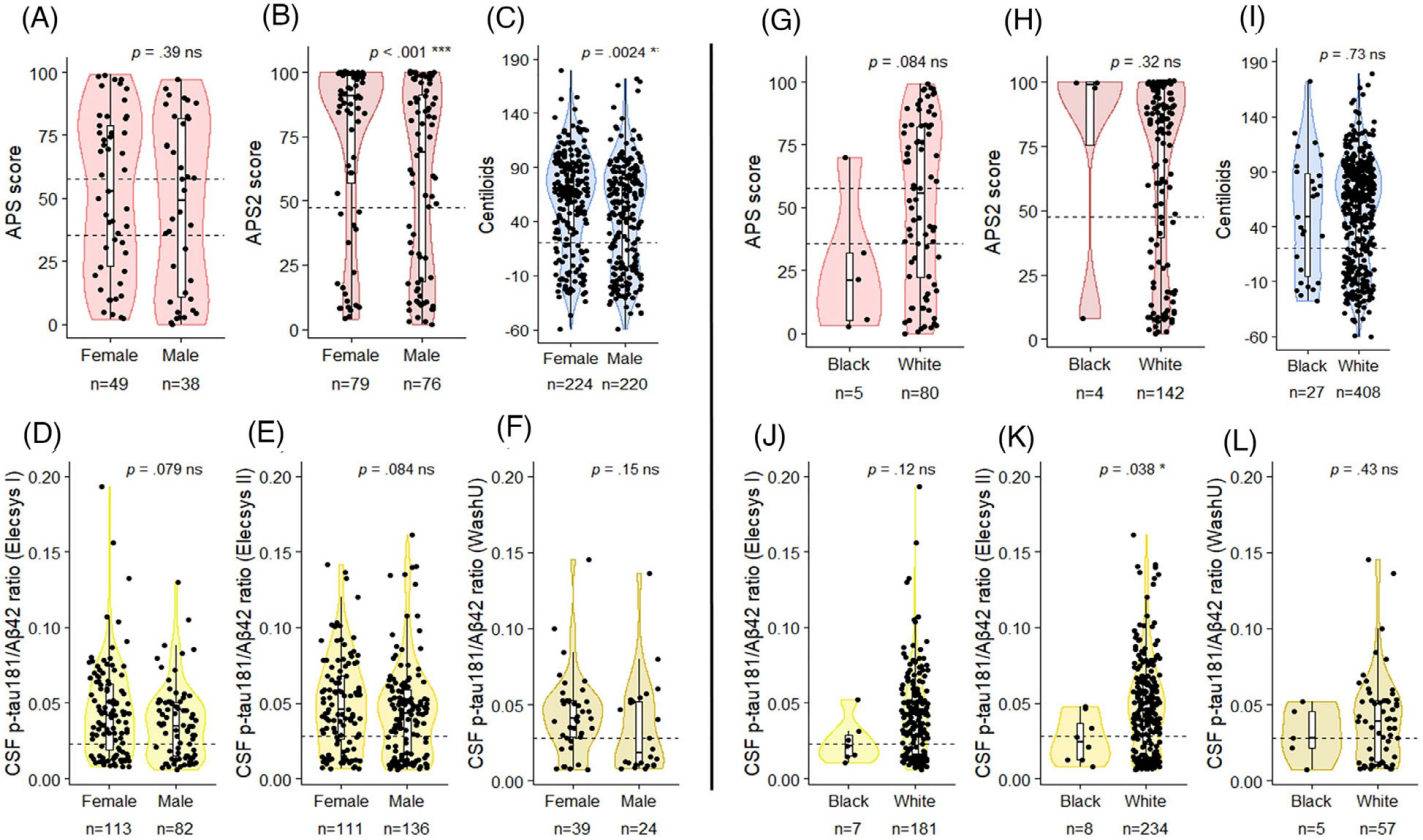
Absolute values of AD biomarker testing according to sex, race, and test version. APS2 score in blood (A, B, G, H), amyloid PET CL values (C, I), and CSF p‐tau181/Aβ42 ratio (D–F, J–L), stratified by sex (left) and race (right). Violin box‐plots representing median, interquartile range, and distribution. The dashed lines indicate the positivity threshold values according to the test version used, that is, 0.023 for Elecsys I, 0.028 for Elecsys II, 20CL for the CL scale, and 47.5 for the APS2 score; the PrecivityAD blood test has two thresholds (35.5 and 57.5) for the APS score and an intermediate range. Note that CL values were not available for every individual. A Kruskal–Wallis test was used for group comparisons. Aβ, amyloid beta; APS, amyloid probability score; CL, Centiloid; CSF, cerebrospinal fluid; PET, positron emission tomography.

The rates of biomarker positivity were 70% across all three modalities (Tables S3 and S4). Women were more likely than men to be AD biomarker positive across all modalities (*p *= 0.001, Table S3), which was statistically significant for amyloid PET visual read (*p *= 0.04) and CSF tests (*p *= 0.04) (Figure 3), but not for the less commonly used blood tests. However, for the PrecivityAD2 blood test, women had higher absolute APS2 scores compared to men (*p *< 0.001, Figure 4A, B). In addition, women had higher amyloid PET CL values compared to men (*p *= 0.002, Figure 4C), but not for the CSF p‐tau181/Aβ ratio, when stratified by different test versions (Figure 4D–F). White individuals were more likely than Black individuals to be AD biomarker positive (*p *< 0.01, Table S4), which was statistically significant for CSF tests (*p *< 0.01), although the total number of Black individuals in the cohort was low (*n* = 53, 4.6%). In exploratory analyses, continuous values for blood tests or amyloid PET did not vary significantly by race (Figure 4G–L).

We also compared comorbidity rates by sex (Table S3) and race (Table S4). Men were diagnosed with hypertension (*p* = 0.02), hyperlipidemia (*p* = 0.03), diabetes (*p* < 0.01), and myocardial infarction (*p* < 0.01) more frequently than women. Black patients had diabetes (*p* < 0.001), cerebrovascular disease (*p* = 0.06), and CKD (*p* = 0.03) more frequently than White patients.

### 3.6 Biomarker positivity predicted by demographic and clinical factors

A logistic regression analysis was performed with biomarker status (by amyloid PET, CSF, or blood testing), as the outcome and age, sex, race and presence of hypertension, diabetes, or CKD as predictors (Table 2). For individuals with more than one biomarker test, the last test was considered. Increasing age in years was associated with an increased probability of amyloid positivity (odds ratio [OR] 1.05, 95% confidence interval [CI] 1.03–1.07, *p* < 0.001). Female patients were more likely to be biomarker positive than male patients (OR 1.48, 95% CI 1.11–1.98, *p* < 0.01). White individuals were more likely to be biomarker positive compared to Black patients (OR 1.95, 95% CI 1.03–3.68, *p* = 0.04). The presence of either hypertension (OR 0.64, 95% CI 0.46–0.89, *p* < 0.01) or diabetes (OR 0.54, 95% CI 0.38–0.77, *p* < 0.001) reduced the likelihood of biomarker positivity. In this model that included demographic variables and other comorbidities, CKD had a trend towards influencing the likelihood of biomarker positivity (*p* = 0.051).

**TABLE 2 alz71442-tbl-0002:** Logistic regression model for biomarker status.

Parameter	Estimate	*SE*	Odds ratio	95% CI (OR)	*p‐*value
Intercept	−2.67	0.75	0.07	0.02–0.30	<0.001
Age	0.04	0.01	1.05	1.03–1.07	<0.001
Sex, female	0.39	0.15	1.48	1.11–1.98	<0.01
Race, White	0.67	0.33	1.95	1.03–3.68	0.04
Hypertension	−0.44	0.17	0.64	0.46–0.89	<0.01
Diabetes	−0.61	0.18	0.54	0.38–0.77	<0.001
CKD	−0.45	0.23	0.64	0.41–1.00	0.051

*Note*: The last biomarker test performed (positive or negative by amyloid PET, CSF, or blood test) was considered as outcome and age, sex, and race as well as presence of hypertension, diabetes, or CKD as predictors. Only Black and White patients were included in this analysis. For the blood tests, only PrecivityAD2 test results were included.

Abbreviations: CI, confidence interval; CKD, chronic kidney disease; CSF, cerebrospinal fluid; PET, positron emission tomography; SE, standard error.

### 3.7 Sensitivity analyses

We performed additional analyses to examine whether the severity of cognitive impairment or interactions with sex or race affected the results. We included the severity of cognitive impairment in the logistic regression model (CDR ≤0.5 or CDR >0.5), and it was not a significant predictor, suggesting that the severity of cognitive impairment in memory clinic patients selected for biomarker testing was not strongly associated with the presence of AD pathology. When the interactions of sex with hypertension and diabetes were included, the interactions were not significant. However, in these models, the main effects for sex and hypertension lost significance, whereas the main effect for diabetes remained significant (Table S5). When the interactions of race with hypertension and diabetes were included, both interactions were significant (*p* = 0.045 for race x hypertension and *p* = 0.04 for race *x* diabetes). The main effects for race and diabetes remained significant, whereas the main effect for hypertension lost significance (Table S6). When hyperlipidemia and cerebrovascular disease were included, neither was statistically significant and the overall significance of the previous predictors did not change (Table S7).

## 4 DISCUSSION

The[Table alz71442-tbl-0002] objective of this study was to evaluate the use of different AD biomarker modalities (amyloid PET scans, CSF tests, and blood tests) between June 2021 and March 2025. Over this period, which included the date of traditional FDA approval for lecanemab (July 2023), the WashU MDC increased biomarker testing approximately sevenfold. Higher rates of biomarker positivity were associated with older age, female sex, and White race, whereas lower rates were associated with hypertension and diabetes. Rates of biomarker positivity were ≈70% across the three biomarker modalities, which is higher than found in previous studies of memory clinics that were focused on patients with high diagnostic uncertainty.^41^ This likely reflects that at WashU MDC, many patients with a high clinical suspicion of symptomatic AD underwent biomarker testing to evaluate eligibility for ATTs.

In 2021, the MDC performed approximately seven CSF tests per month. In February 2022, blood tests started being used. After the Centers for Medicare & Medicaid Services began reimbursing for amyloid PET in October 2023, the proportion of biomarker testing with amyloid PET markedly increased. By July 2024, ∼50 biomarker tests per month were performed, with the majority being amyloid PET. Similar trends in biomarker modality use were recently described by another center.^42^

Age and severity of cognitive impairment were associated with the biomarker modality used. Older age was associated with use of blood tests and amyloid PET, potentially because older patients are more likely to have Medicare, which covers initiation of ATTs based on blood tests and amyloid PET.^3^ Less severely impaired patients were more likely to undergo PET, potentially reflecting frequent use of PET in patients with early symptomatic AD who were being evaluated for ATTs. Sex and race were not associated with the biomarker modality used. Notably, in some research studies, Black individuals were less likely to undergo lumbar puncture compared to Caucasian patients,^43^ but the relatively small number of Black patients with biomarker testing may provide insufficient statistical power for a valid comparison across modalities.

When combined across the three biomarker modalities, females had a 1.5‐fold higher rate of biomarker positivity compared to males, and they had higher absolute biomarker values for blood tests and amyloid PET scans. Female sex remained a significant independent predictor for biomarker positivity across modalities, even after adjusting for age and key comorbidities (hypertension, diabetes, and CKD). This is consistent with a previous report of higher amyloid PET positivity rates and pathology burden in females compared to males in a community‐based cohort,^17^ but is in contrast to previous studies that have described higher levels of different blood p‐tau species in men compared to women.^16^, ^18^ Whereas higher rates of biomarker positivity may be explained by the increased prevalence of AD in women compared to men, the mechanisms behind higher pathology burden in females may be multifactorial and complex.^44^

White individuals had twice the likelihood of biomarker positivity when combined across the three modalities compared to Black individuals, even after adjusting for demographic factors and comorbidities, which is consistent with some previous studies.^25^, ^30^, ^31^, ^32^, ^33^ Paradoxically, studies have shown a higher prevalence of cognitive impairment in Black and Hispanic populations, which may suggest a higher proportion of dementia cases being caused by non‐AD etiologies such as cerebrovascular disease.^45^, ^46^, ^47^, ^48^, ^49^ Notably, the number of Black individuals undergoing AD biomarker testing in this study is low, making this finding uncertain and requiring confirmation in larger studies.

The overall prevalence of comorbidities was similar to that reported for other cohorts of AD patients.^50^, ^51^ Notably, hypertension and diabetes were independently associated with a lower risk of biomarker positivity. Because men and Black patients had a significantly higher rate of certain comorbidities, we also performed sensitivity analyses examining potential interactions between sex or race with hypertension or diabetes. When the interaction of sex or race with hypertension was included, the main effect of hypertension on biomarker status lost significance. However, diabetes continued to have a main effect on biomarker status even when the interaction of sex or race was included. The lower risk of AD biomarker positivity associated with hypertension and diabetes seems inconsistent given that certain cardiovascular risk factors increase the risk for AD,^52^ as well as results from another recent study describing significant associations between the presence of particularly diabetes or hypertension and different AD plasma biomarker levels.^53^ However, these comorbidities may be more strongly associated with non‐AD causes of cognitive impairment (e.g., vascular dementia).

When CKD was included in the model with hypertension and diabetes, we saw only a trend toward an effect on the likelihood of biomarker positivity. CKD has been associated with higher AD blood biomarker levels in other studies,^54^, ^55^ although the PrecivityAD2 test may be less susceptible to these effects.^56^, ^57^ In addition, an effect may not have been observed because multiple biomarker modalities were included in the model and/or because of inaccurate information on CKD in the electronic health record.

This study has some limitations. Few patients from our cohort (6%) underwent AD biomarker testing with more than one modality. In addition, the number of Black patients who underwent biomarker testing was relatively small (*n* = 53), particularly for patients with blood tests, thereby limiting statistical power. We have reported previously that Black patients are underrepresented in the MDC clinic, with only 11% of MDC patients self‐identifying as Black compared to an estimated catchment area including 16% Black individuals.^37^ This suggests a disproportionately low rate of biomarker testing in Black patients (5%) for reasons that are not yet understood. However, we have found that Black patients are more likely to have moderate or severe dementia at their first MDC visit,^37^ ^]^ that is, when they are no longer eligible for ATTs; therefore, they may be less likely to undergo biomarker testing to confirm amyloid pathology. To maximize statistical power, we combined different biomarker modalities for analysis. If the cohort had greater statistical power, different effects of demographic factors and comorbidities could potentially be present for separate biomarker modalities or even specific tests.

In summary, in a large U.S. specialty memory clinic, AD biomarker testing dramatically increased following the FDA approval of lecanemab. In this clinic, AD blood biomarker testing is used to confirm amyloid pathology and initiate lecanemab treatment. Overall, we report high rates of AD biomarker positivity across biomarker modalities and found associations with age, sex and race. These results may inform estimates of amyloid pathology in clinical practice and should be further investigated in larger, more diverse populations.

## CONFLICT OF INTEREST STATEMENT

AH has received research funding from Novo Nordisk and collaborated with Biogen on a research project. MP reported personal honoraria from Eisai and Eli Lilly for participating in scientific board meetings and educational activities. She has served as a sub‐investigator on sponsored clinical trials from Eisai and Eli Lilly outside the submitted work. AG and WJBP have received research funding from Novo Nordisk. MRP received Avid Radiopharmaceuticals, Inc. and Lantheus Medical Imaging, Inc., consulting fees outside of the submitted work. RJB co‐founded C2N Diagnostics. Washington University (WashU) and RJB has equity ownership interest in C2N Diagnostics and receive royalty income based on technology (stable isotope labeling kinetics, blood plasma assay, and methods of diagnosing Alzheimer's disease with phosphorylation changes) that is licensed by WashU to C2N Diagnostics. RJB receives income from C2N Diagnostics for serving on the scientific advisory board. RJB has received research funding from Avid radiopharmaceuticals, Janssen, Roche/Genentech, Eli Lilly, Eisai, Biogen, AbbVie, Bristol Myers Squibb, and Novartis. TLSB reported grants from Siemens; loan of equipment from Hyperfine; precursors and technology transfer from Lantheus Radiopharmaceutical and Eli Lilly/Avid Radiopharmaceuticals; personal fees from Biogen, Eli Lilly, Eisai, Bristol Myers Squibb, Johnson & Johnson, and Merck; and payment for generation of medical education activities from Medscape, PeerView, and Neurology Today, outside the submitted work. JCM reported grants from the National Institutes of Health during the conduct of the study. BJS reported advisory board fees and grants from Eisai and Eli Lilly, outside the submitted work. SES has not received any financial compensation from pharmaceutical or diagnostics companies since 2024, when she received honoraria for serving on scientific advisory boards on biomarker testing and education for Eisai and Novo Nordisk and speaking fees from Eisai, Eli Lilly, and Novo Nordisk. She has received honoraria for educational presentations from Medscape, PeerView, and the Academy for Continued Healthcare Learning. She has provided unpaid scientific advising to Acumen, Biogen, Cognito Therapeutics, Danaher, Eisai, Eli Lilly, Johnson and Johnson Innovative Medicine, and Sanofi. All other authors report no declarations. Author disclosures are available in the Supporting Information.

## CONSENT STATEMENT

Patient consent was not necessary for this study.

## Supporting information




Supporting Information

Supporting Information

Supporting Information

Supporting Information

Supporting Information

Supporting Information

Supporting Information

Supporting Information
