# Supplementary material for: Usage and positivity rates of Alzheimer's disease biomarkers in a memory clinic
Source: Alzheimers Dement. 2026 May 4;22(5):e71442. doi: 10.1002/alz.71442 (PMC13137296; doi:10.1002/alz.71442)
Supplement: Supplementary file 2 — Supporting Information [file ALZ-22-e71442-s004.docx]

**Supplementary Table 1:** **Characteristics of patients undergoing a single AD biomarker test stratified by different modalities.**

| **Characteristic** | **n** | **Blood (n = 203)** | **n** | **CSF (n = 456)** | **n** | **PET (n = 414)** | ***P*** **value** |
| --- | --- | --- | --- | --- | --- | --- | --- |
| **Age (years)** | 203 | 75.4 (71.0-79.6) | 456 | 71.8 (65.2-77.0) | 414 | 73.8 (69.3-78.5) | <.0001 |
| **Sex (female)** | 203 | 109 (53.7%) | 456 | 239 (52.4%) | 414 | 211 (51.0%) | .80 |
| **Race (Black/White/Other)** | 197 | 8/185/4 | 450 | 18/427/5 | 412 | 24/384/4 | .54 |
| **Ethnicity (non-Hispanic)** | 195 | 192 (98.5%) | 447 | 440 (98.4%) | 406 | 404 (99.5%) | .29 |
| **Lecanemab treatment** | 203 | 44 (21.7%) | 456 | 126 (27.6%) | 414 | 85 (20.5%) | .04 |
| **BMI** **(kg/m²)** | 106 | 25.9 (22.8-29.9) | 231 | 25.9 (22.8-29.8) | 178 | 26.5 (23.2-29.7) | .79 |
| **Hypertension** | 196 | 136 (69.4%) | 450 | 272 (60.4%) | 363 | 231 (63.6%) | .09 |
| **Hyperlipidemia** | 196 | 130 (66.3%) | 450 | 271 (60.2%) | 363 | 228 (62.8%) | .33 |
| **Diabetes** | 196 | 40 (20.4%) | 450 | 89 (19.8%) | 363 | 73 (20.1%) | .98 |
| **Cerebrovascular disease** | 196 | 51 (26.0%) | 450 | 72 (16.0%) | 363 | 71 (19.6%) | .01 |
| **Myocardial infarction** | 196 | 10 (5.1%) | 450 | 17 (3.8%) | 363 | 24 (6.6%) | .19 |
| **Chronic kidney disease** | 196 | 31 (15.8%) | 450 | 41 (9.1%) | 363 | 41 (11.3%) | .046 |
| **Liver cirrhosis** | 196 | 1 (0.5%) | 450 | 1 (0.2%) | 363 | 3 (0.8%) | .48 |
| **Polyneuropathy** | 196 | 21 (10.7%) | 450 | 39 (8.7%) | 363 | 39 (10.7%) | .55 |
| **Creatinine** **(mg/dl)** | 84 | 0.9 (0.8-1.1) | 185 | 0.9 (0.8-1.2) | 175 | 0.91 (0.81-1.12) | .45 |
| **GFR (****mL/min/1.73m²)** | 74 | 75.0 (58.0-85.0) | 165 | 73.0 (60.0-85.0) | 154 | 75 (62-86) | .75 |
| **HbA1c (%)** | 32 | 5.8 (5.5-6.2) | 60 | 5.8 (5.4-6.2) | 69 | 5.9 (5.5-6.8) | .43 |
| **LDL (mg/dl)** | 49 | 66.0 (56.0-85.0) | 117 | 86.0 (62.0-112.0) | 114 | 86 (67-116) | .003 |
| **Triglycerides (mg/dl)** | 52 | 94.0 (67.0-132.0) | 122 | 99.0 (74.0-138.0) | 113 | 89 (67-137) | .52 |
| **MMSE score** | 187 | 24 (21-27) | 434 | 24 (21-26) | 343 | 25 (23-27) | <.0001 |
| **CDR global score (0/0.5/≥1)** | 187 | 9/130/48 | 421 | 6/317/98 | 319 | 7/273/39 | <.0001 |

NOTE. Age, laboratory values, BMI and cognitive scores are selected from the time of biomarker testing. Some individuals did not identify as Black or White. Information on Ethnicity, BMI, comorbidities, laboratory values and cognition were not available for every individual; the “n” columns indicate the number of patients for which the data were available. Counts and percentages are provided for categorical variables. Median and interquartile range are indicated for continuous variables. A Chi-square test was calculated for categorial variables and a Kruskal-Wallis test for continuous variables. BMI: Body Mass Index, GFR: Glomerular Filtration Rate, LDL: Low-Density Lipoprotein, MMSE: Mini Mental State Examination, CDR: Clinical Dementia Rating.
