# Supplementary material for: Usage and positivity rates of Alzheimer's disease biomarkers in a memory clinic
Source: Alzheimers Dement. 2026 May 4;22(5):e71442. doi: 10.1002/alz.71442 (PMC13137296; doi:10.1002/alz.71442)
Supplement: Supplementary file 4 — Supporting Information [file ALZ-22-e71442-s006.docx]

**Supplementary Table 3: Biomarker results and comorbidity counts stratified by sex.**

| **Biomarker** | **All (n = 1,136)** | **Female (n = 590)** | **Male (n = 546)** | ***P* value** |
| --- | --- | --- | --- | --- |
| **Overall AD biomarker positivity** | 750/1,065 (70.4%) | 413/552 (74.8%) | 337/513 (65.7%) | .001 |
| **CSF p-tau181/Aβ42 positivity rate** | 348/505 (68.9%) | 192/263 (73.0%) | 156/242 (64.5%) | .04 |
| **Elecsys I Mayo positivity rate** | 137/195 (70.3%) | 82/113 (72.6%) | 55/82 (67.1%) | .41 |
| **Elecsys II Mayo positivity rate** | 171/247 (69.2%) | 81/111 (73.0%) | 90/136 (66.2%) | .25 |
| **Elecsys II WashU positivity rate** | 40/63 (63.5%) | 29/39 (74.4%) | 11/24 (45.8%) | .02 |
| **Elecsys I Mayo value** | 0.037 (0.016-0.054) | 0.041 (0.018-0.063) | 0.035 (0.015-0.05) | .08 |
| **Elecsys II Mayo value** | 0.044 (0.019-0.062) | 0.046 (0.025-0.068) | 0.043 (0.016-0.059) | .08 |
| **Elecsys II WashU value** | 0.039 (0.012-0.052) | 0.041 (0.028-0.052) | 0.016 (0.011-0.052) | .15 |
| **Amyloid PET positivity rate** | 325/455 (71.4%) | 175/231 (75.8%) | 150/224 (67.0%) | .04 |
| **Centiloids** | 63.9 (11.3-88.2) | 67.7 (28.7-92.7) | 53.0 (-1.8-82.7) | <.01 |
| **PrecivityAD2 positivity rate** | 108/155 (69.7%) | 60/79 (76.0%) | 48/76 (63.2%) | .08 |
| **APS2** | 85 (30-99) | 91 (53-100) | 68 (18-91) | <.001 |
| **PrecivityAD (low/intermediate/high)** | 31/14/42 | 17/8/24 | 14/6/18 | .98 |
| **APS** | 53 (17-80) | 50 (21-79) | 45 (10-80) | .39 |
| **Hypertension** | 673/1,070 (62.9%) | 328/551 (59.5%) | 345/519 (66.5%) | .02 |
| **Hyperlipidemia** | 667/1,070 (62.3%) | 326/551 (59.2%) | 341/519 (65.7%) | .03 |
| **Diabetes** | 213/1,070 (19.9%) | 92/551 (16.7%) | 121/519 (23.3%) | <.01 |
| **Cerebrovascular disease** | 198/1,070 (18.5%) | 103/551 (18.7%) | 95/519 (18.3%) | .87 |
| **Myocardial infarction** | 58/1,070 (5.4%) | 20/551 (3.6%) | 38/519 (7.3%) | <.01 |
| **CKD** | 119/1,070 (11.1%) | 53/551 (9.6%) | 66/519 (12.7%) | .11 |
| **Liver cirrhosis** | 5/1,070 (0.5%) | 2/551 (0.4%) | 3/519 (0.6%) | .61 |
| **Polyneuropathy** | 103/1,070 (9.6%) | 54/551 (9.8%) | 49/519 (9.4%) | .84 |

NOTE. Shown are blood, CSF and amyloid PET positivity rates and absolute values for all different test versions in use as well as comorbidity counts stratified by sex. Note that information on comorbidities is not available for every individual. The overall AD biomarker positivity includes PrecivityAD2 for blood tests only. Note that 63 patients underwent multiple biomarker testing. For the overall biomarker positivity count, only the last test was included. Counts and percentages are provided for categorical variables. Median and interquartile range are indicated for continuous variables. A Chi-square test was calculated for categorial variables and a Kruskal-Wallis test for continuous variables. CKD, Chronic kidney disease.
