# Supplementary material for: Usage and positivity rates of Alzheimer's disease biomarkers in a memory clinic
Source: Alzheimers Dement. 2026 May 4;22(5):e71442. doi: 10.1002/alz.71442 (PMC13137296; doi:10.1002/alz.71442)
Supplement: Supplementary file 6 — Supporting Information [file ALZ-22-e71442-s005.docx]

**Supplementary Table 5: Logistic regression including sex interactions.** The last biomarker test performed (positive or negative by amyloid PET, CSF or blood test) was considered as outcome and age, sex, race as well as presence of hypertension, diabetes or chronic kidney disease as well as the interactions between sex and hypertension or diabetes as predictors.

| **Parameter** | **Estimate** | **SE** | **Odds ratio** | **95% CI (OR)** | ***P* value** |
| --- | --- | --- | --- | --- | --- |
| **Intercept** | -2.71 | 0.76 | 0.07 | 0.02 – 0.30 | < .001 |
| **Age** | 0.04 | 0.01 | 1.05 | 1.03 – 1.07 | < .001 |
| **Sex (female)** | 0.46 | 0.26 | 1.56 | 0.96 – 2.62 | .07 |
| **Race (White)** | 0.67 | 0.33 | 1.96 | 1.03 – 3.71 | 0.04 |
| **Hypertension** | -0.37 | 0.22 | 0.69 | 0.44 – 1.07 | .10 |
| **Diabetes** | -0.67 | 0.23 | 0.51 | 0.32 – 0.81 | < .01 |
| **Chronic kidney disease** | -0.45 | 0.23 | 0.64 | 0.41 – 1.00 | .051 |
| **Sex (female)*Hypertension** | -0.15 | 0.32 | 0.86 | 0.46 – 1.61 | .63 |
| **Sex (female)*Diabetes** | 0.14 | 0.36 | 1.15 | 0.57 – 2.33 | .69 |

NOTE. Only Black and White patients were included in this analysis. For the blood tests, only PrecivityAD2 test results were included. SE: standard error.
